# Using biological networks to integrate, visualize and analyze genomics data

**DOI:** 10.1186/s12711-016-0205-1

**Published:** 2016-03-31

**Authors:** Theodosia Charitou, Kenneth Bryan, David J. Lynn

**Affiliations:** EMBL Australia Group, Infection and Immunity, South Australian Health and Medical Research Institute (SAHMRI), North Terrace, Adelaide, SA 5000 Australia; Systems Biology Ireland, University College Dublin, Belfield 4, Ireland; Teagasc, The Agriculture and Food Development Authority, Co Meath, Ireland; School of Medicine, Flinders University, Bedford Park, SA 5042 Australia

## Abstract

Network biology is a rapidly developing area of biomedical research and reflects the current view that complex phenotypes, such as disease susceptibility, are not the result of single gene mutations that act in isolation but are rather due to the perturbation of a gene’s network context. Understanding the topology of these molecular interaction networks and identifying the molecules that play central roles in their structure and regulation is a key to understanding complex systems. The falling cost of next-generation sequencing is now enabling researchers to routinely catalogue the molecular components of these networks at a genome-wide scale and over a large number of different conditions. In this review, we describe how to use publicly available bioinformatics tools to integrate genome-wide ‘omics’ data into a network of experimentally-supported molecular interactions. In addition, we describe how to visualize and analyze these networks to identify topological features of likely functional relevance, including network hubs, bottlenecks and modules. We show that network biology provides a powerful conceptual approach to integrate and find patterns in genome-wide genomic data but we also discuss the limitations and caveats of these methods, of which researchers adopting these methods must remain aware.

**Electronic supplementary material:**

The online version of this article (doi:10.1186/s12711-016-0205-1) contains supplementary material, which is available to authorized users.

## Background

Cellular processes are controlled and coordinated at multiple levels by tightly regulated transcriptional, post-transcriptional and post-translational molecular networks. Recent advances and falling costs of technologies such as next-generation sequencing (NGS) and mass spectrometry (MS) are enabling researchers to catalogue the component molecules of these networks at a genome-wide scale and under a large number of different experimental conditions (e.g. time points, cell types, stimuli and treatments). These high-throughput approaches typically result in one or more lists of genes or proteins (or other molecules such as lipids or metabolites) that are significantly altered, in their expression for example, at a specific time-point or condition. However, without further analysis, such lists are often of relatively limited use and fail to reveal the complex inter-relationships that may exist between molecules, their coordinated functions, and the emergent properties of the system. In this review, we discuss how researchers can move from gene lists to more systems-oriented analyses of their data, with a particular focus on using experimentally-supported molecular interaction networks. We discuss how to use publicly available bioinformatics tools and molecular interaction data to construct a network from a gene/protein list and explore how to subsequently visualize and analyze these networks for the purpose of revealing new insights into the phenotype of interest at the systems’ level. We give examples of how such approaches are being applied in the literature and we will focus particularly on examples of relevance to the animal functional genomics community.

## Gene ontology and pathway analysis

As discussed above, the initial output of most genome-wide ‘omics’ experiments is a list of genes (or their products) that are significantly altered in the condition of interest. Typically, the first step in the investigation of these datasets is a functional enrichment analysis, which determines whether the list of genes is statistically enriched for certain biological processes or functions. The Gene Ontology (GO) consortium, for example, provides a controlled hierarchical vocabulary of terms for describing genes and their encoded products in terms of their molecular functions, biological processes or cellular components [1]. A GO enrichment analysis can be undertaken using one of the many publicly available tools (http://geneontology.org/page/go-enrichment-analysis) and these analyses examine the gene list for the occurrence of GO terms that are more prevalent in the query gene list than expected by chance (it is important to note that using an appropriate background or ‘universe’ to assess statistical significance is essential) [2]. Such over-represented terms may highlight previously unrecognised biological processes (as opposed to individual genes) that are preferentially and differentially regulated in the condition of interest. A feature of GO that is both a strength and a limitation is its hierarchical structure. Although efforts have been made to account for this structure in GO enrichment analyses [3], it can still be difficult to determine which level of the hierarchy is most responsible for the statistical enrichment. Often the most enriched terms are broad functional categories which can be of limited use to inform new functional insight.

In cells, biological pathways are the biochemical engines that are responsible for the transduction of signals (often received by receptors) into output responses (e.g. activation of a transcription factor and downstream gene expression). An enrichment analysis based on pathway annotations can therefore contain information that is more directly relevant and interpretable regarding the important processes at play in a particular condition. A wide variety of pathway analysis methods are available [4], including over-representation methods such as those implemented in KEGG [5], Reactome [6], WikiPathways [7, 8], InnateDB [9], or DAVID [10]; more quantitative methods based on gene set enrichment [11]; and more recent methods that attempt to account for the fact that not all genes have the same power to distinguish between different pathways [12].

Although powerful, pathway analysis methods also have their limitations. First, the majority of genes have not been assigned to a canonical pathway (e.g., more than 85 % of human *Ensembl* genes are not mapped to any KEGG pathway), and second, for those that are, there is a heavy bias towards well-studied signalling pathways [13]. Thus, pathway analysis can tell us a lot about what we already know but less about new and unexpected relationships between genes of interest or indeed between the pathways themselves.

## Network analysis

Network biology is a rapidly developing area of research, which recognises that biological processes are not chiefly controlled by individual proteins or by discrete, unconnected linear pathways, but rather by a complex system-level network of molecular interactions [14]. Understanding how these molecular interaction networks give rise to emergent biological processes and identifying the important nodes and other topological features, which are key to controlling them, are crucial to understanding complex phenotypes in health and disease. Network medicine theory also proposes that disease-associated phenotypes are not the result of single gene mutations acting in isolation but are rather due to the perturbation of a gene’s network context [15]. Therefore, the elucidation of disease mechanisms and the development of effective therapeutic targets require a deep understanding of how molecular interaction networks are pathogenically dysregulated. In practice, network analysis can also be an extremely powerful and complementary approach to traditional enrichment analysis methods [16]. Advantages of this approach include the fact that network-based analyses are both more data driven and also less constrained by the limits of current functional annotations, as proteome-scale maps of the interactome (the complete complement of molecular interactions within a biological system) are now available for several species, including humans [17]. Because of this, network analyses are less biased towards well-studied pathways and have a far greater coverage of known genes and proteins.

The interactome may be intuitively represented and interpreted by constructing a graph or network, in which an entity (e.g. gene, transcript, protein, miRNA, or metabolite) is represented by a *node* and its relationships or interactions to other entities by a series of pairwise lines or *edges* between these nodes. Networks are not restricted to one type of entity (node type) or relationship (edge type) and are often used to visualize and interpret several types of molecules and their molecular relationships simultaneously (physical interaction, reaction, regulation, correlation, etc.). This allows a more complete and realistic representation of a biological system. Additional information associated with the nodes (e.g. gene expression data) or edges (e.g. a confidence score) can also be easily integrated via the use of node and edge attributes. Another advantage is that network/graph theory and supporting computational methods are well established in other domains, which has allowed for the rapid re-purposing and development of software to support network visualization and analysis in biology [18].

There are two broad approaches that one can adopt when performing network analysis on a gene list of interest. The first is to overlay the genome-wide ‘omics’ data (e.g. gene expression data) on a pre-established global network of experimentally-supported interactions (e.g. public protein–protein interactions (PPI)), while the second is to infer a network directly from the data generated in the experiment (for a review of these approaches see [19]). In this review, we focus largely on the former integrative method and discuss both the strengths and limitations of this approach.

## Constructing a molecular interaction network from a list of genes

The first consideration when constructing a molecular interaction network from publicly available data is what type of interaction data one wants to include in the network and where to source that data. A sometimes confusing plethora of molecular interaction databases are publicly available [20]. Researchers need to be aware that not all of these databases contain the same type or quality of interaction data. Some databases, such as those that are members of the International Molecular Exchange (IMEx) Consortium [21], promote painstaking manual curation of experimentally-validated interaction data directly from the peer-reviewed biomedical literature. Other so-called ‘meta’-databases integrate and repackage interaction data from these primary sources and make it available through a single portal. Some databases also supplement experimentally-validated interaction information with computationally-predicted interactions [22]. Although this practice is useful for enriching a sparse experimental interaction network, users need to be more aware of this. We also suggest that researchers compare results that are generated using an experimentally-validated network versus the network that has been supplemented with computationally-predicted edges. Researchers should also note that primary interaction databases show limited overlap in the interaction information they provide. This is partly intentional, as developers of the IMEx databases take steps to avoid duplication of effort in their very labour-intensive manual curation processes. However, this also means, that a lot of additional well-supported public interaction information will be ignored if interactions are sourced from one database only. Fortunately, web-services, such as PSICQUIC [23], are available to enable researchers to query multiple databases simultaneously, although, to date, the majority of papers reporting network analyses have not been so comprehensive.

Once all the experimentally-validated interactions that involve a given gene list (or their products) have been retrieved, there are some additional points to consider before proceeding to the downstream network analysis. First, the experimentally-validated interactions retrieved may be of several types, including physical (e.g. PPI or protein-DNA), regulatory (e.g. microRNA-mRNA), or biochemical interactions (e.g. phosphorylations). Although it may be valuable to integrate many types of interactions, one must proceed with caution since the meaning of an edge in such a network will vary substantially and this needs to be taken into account during subsequent analyses of the data. Physical PPI, for example, are usually undirected edges and may capture information regarding protein complexes, whereas biochemical interactions are usually directed (e.g. A phosphorylates B) and relate to a flow of signal information. Another consideration in the case of physical protein interactions that are determined by affinity purification coupled with mass spectrometry (AP-MS) [24], is that these methods usually cannot distinguish between direct and indirect interactors, although they are often represented as direct binary interactions in networks that are constructed using publicly available tools.

Another important consideration is the level of confidence associated with a particular molecular interaction, which may vary considerably, depending on how that interaction was experimentally determined. On the one hand, high-throughput approaches such as Yeast 2-Hybrid (Y2H), can be used to generate large amounts of data on the interactome, which are, however, often associated with relatively high false positive and false negative rates [25]. On the other hand, interactions that are curated from more focused low-throughput studies described in the biomedical literature may have greater confidence but they can be biased towards well-studied pathways and biological processes. Several metrics have now been developed to provide an interaction confidence score and these scores can be reflected in networks using edge weights [26].

Finally, one must also bear in mind that the interactome retrieved from databases is a static snapshot of all known possible interactions for the given query list. Many of these interactions will be context-specific (e.g. occurring in a particular cell-type, or under specific conditions; or for a particular isoform of a protein [27]). Unfortunately, there is relatively little high-throughput context-specific interactome data in the literature and, thus, in molecular interaction databases. If analyses were restricted only to interactions that were context-specific (e.g. identified in the same cell-type), most of the data would be discarded. However, researchers can integrate other forms of external contextual information, such as gene or protein expression data, to select the most likely contextual sub-network of nodes and edges.

## Case study: constructing an experimentally-validated molecular interaction network using InnateDB.com

A limitation of using the PSICQUIC web-service to build an interaction network is that it is not particularly accessible for most biologists. Fortunately, there are several more user-friendly web-based platforms available. Here, we provide a case study that describes how to use tools available at InnateDB.com to build and visualize a network of experimentally-validated molecular interactions from a gene list [9, 13]. InnateDB is a comprehensive database that contains more than 300,000 experimentally-validated human, mouse and bovine molecular interactions and more than 3000 pathway annotations, integrated from major public molecular interaction and pathway databases. In addition to this integrated data, the InnateDB curation team has contextually annotated more than 25,000 innate immunity-relevant molecular interactions through their review of more than 5000 biomedical articles. Interactions in InnateDB are curated to MIMIx standards [28] with rich contextual annotations, including the supporting publication, participant molecules, species, interaction detection method, host system, interaction type, cell, cell-line and tissue types, etc., that are associated with each interaction. For more details on InnateDB curation of the innate immunity interactome, see [29]. InnateDB is also an analysis platform that offers seamlessly-integrated, user-friendly bioinformatics tools, including pathway and ontology analysis, network visualization and analysis, and the ability to upload and analyze user-supplied gene expression data (or other forms of quantitative data) in a network and/or pathway context.

It is important to emphasise that InnateDB does not only contain interactions of relevance to innate immunity but, as mentioned above, is also a repository for the entire human and mouse interactomes. The bovine interactome is inferred largely via orthology with human and mouse genes. The limitations of using orthology to infer interlogs is discussed in some detail in [13], but there are few options for researchers working on agriculturally-relevant animal species for which little or no experimentally-validated interactome data is available. It should be noted that the same issues are shared with GO and pathway analyses, as these species-specific annotations have also been largely inferred by orthology. Researchers who work on other mammalian species must map (by orthology) gene identifiers from their species of interest to their corresponding human/mouse gene ID prior to using InnateDB. It is generally not recommended to attempt to infer interlogs from more evolutionarily-distant species, since these interactions are much less likely to be conserved.

### How to build a network using InnateDB.com

**Fig. 1:**
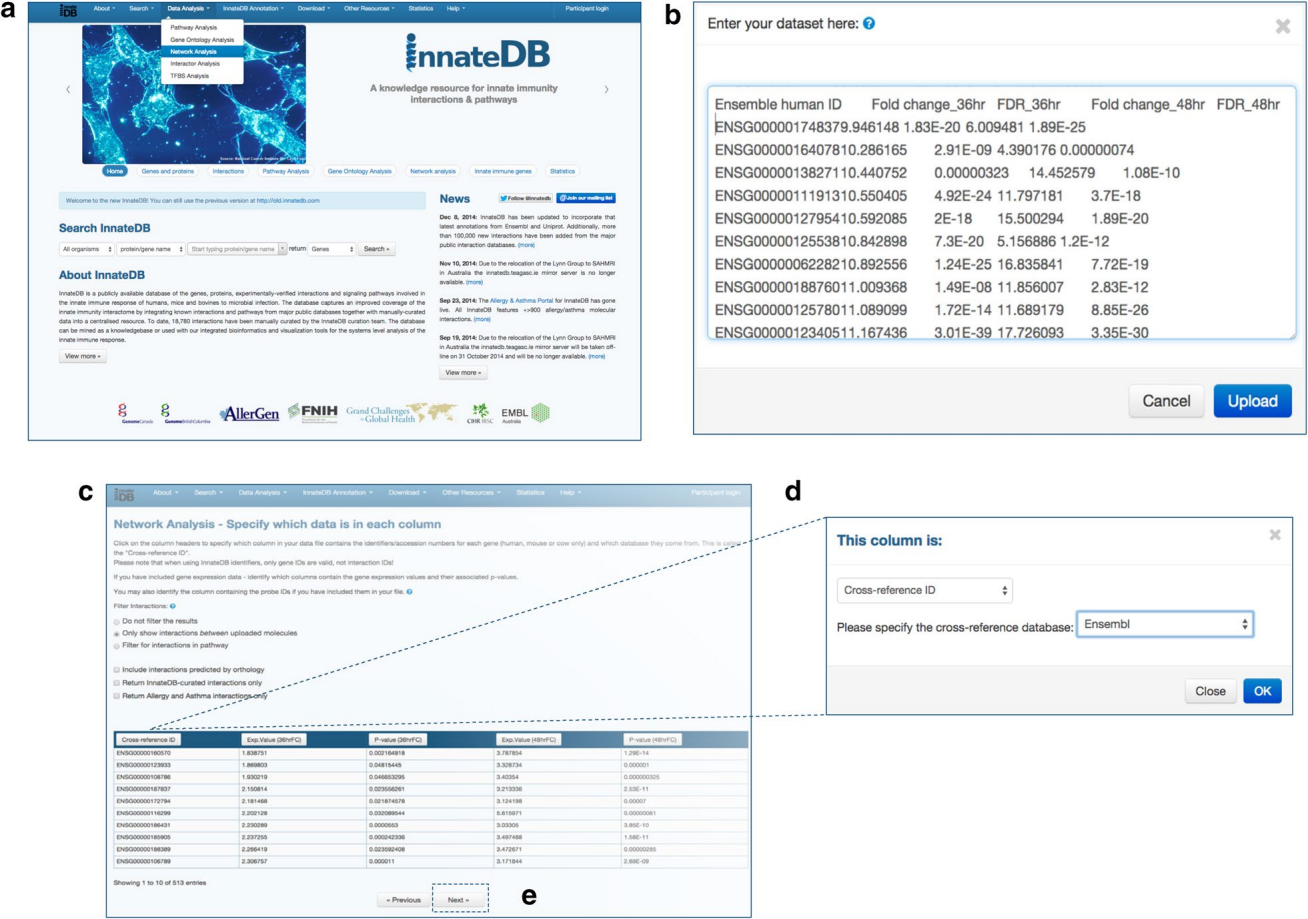
Overview of InnateDB network analysis. **a** Go to the “Data Analysis” menu at the top of the InnateDB.com home page and select “Network Analysis”. **b** Paste a gene list (and any associated quantitative data) into the web form or upload the data via a tab-delimited text file or Excel spreadsheet (.xls files only). **c** Select the options for the network analysis as described in the main text. **d** Click on the column headers to define the columns that contain the gene IDs and the quantitative data. **e** Submit the data by clicking “Next” to tell InnateDB to build the network

Figure 1 outlines how to upload a list of genes (or proteins) and associated quantitative data (e.g. gene expression data) to InnateDB and build, visualize and analyze the molecular interaction network in which these genes (or their encoded products) participate. In this case study, we used a list of 514 genes from Lawless et al. [30] that were found to be significantly up-regulated more than threefold in monocytes isolated from milk at either 36 or 48 h post infection (hpi) with the pathogen *Streptococcus uberis*, which causes mastitis (See Additional file 1: Table S1). Bovine gene ID were mapped to human Ensembl gene ID based on predicted 1:1 orthology, as described in [30]. To perform a network analysis using InnateDB, a user must first go to the “*Data Analysis”* menu at the top of the homepage and select “*Network Analysis*”. The user is then directed to the “*Upload Data*” form, where the gene list can be pasted (as well as any associated quantitative data). Alternatively, these data may be uploaded via a tab-delimited text file or spreadsheet (.xls files only). Quantitative data associated with the genes, which may be measured over as many as 10 different conditions, are incorporated as one or more *nodeattributes* within the subsequent network visualization. InnateDB provides a number of filters to determine which interactions are included in the generated network. The default (“*Do not filter the results*”) will return all the interactions for which at least one participant in the interaction is included in the uploaded list of genes. This is useful to identify nodes in the network that were not detected in the high-throughput experiment from which the input list of genes was derived, but which preferentially interact with those genes/proteins. In the case of a gene expression dataset, such nodes may represent genes that are not differentially regulated at the transcriptional level but are nonetheless critical regulators and important components of the system under study (e.g. transcription factors that are regulated at the post-translational level; genes that fall below the thresholds used to define differentially-expressed genes; or genes that are differentially expressed at a time-point that was not surveyed). The user can also choose the more conservative “*Only show interactions between uploaded molecules*” option, which will return only those interactions for which both interaction participants are members of the uploaded gene list. A third option allows users to return only interactions of relevance to a selected pathway of interest. Users can also choose to include interactions that were predicted by orthology, or only those manually annotated by InnateDB. In general, we would recommend that, unless the dataset is only relevant to innate immunity, interactions from all databases integrated into InnateDB should be returned. Users will then be presented with a table that previews the data that has been uploaded. After the gene ID and quantitative data columns have been defined, by clicking on column headers, the query data can be submitted to InnateDB, which then builds the network.

## Network visualization and download

**Fig. 2:**
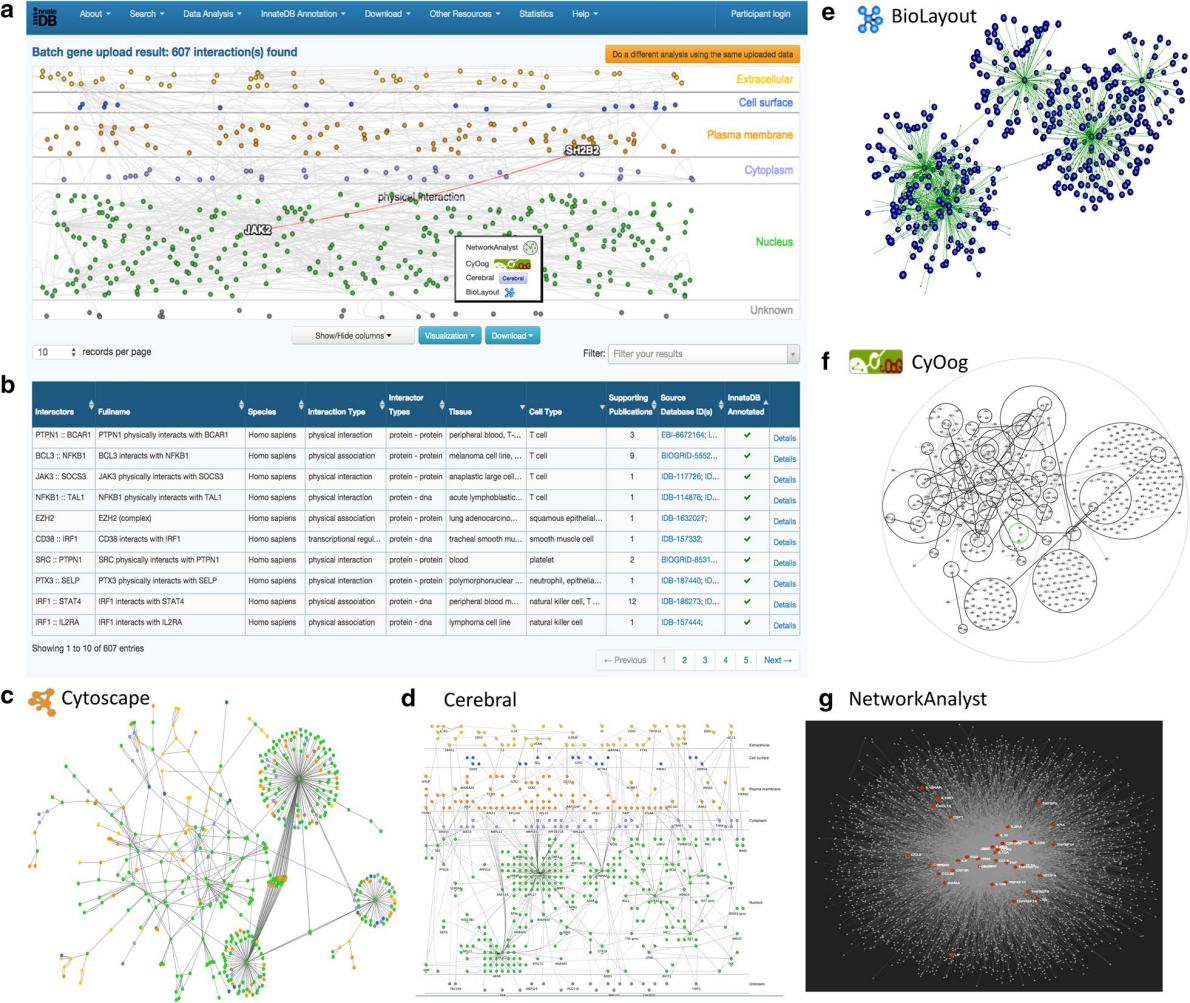
Visualizing the network. InnateDB was used to construct a network of genes that were significantly up-regulated in monocytes isolated from bovine milk at either 36 or 48 h post infection (hpi) with *Streptococcus uberis*. Only experimentally-validated interactions between genes in the uploaded dataset are shown. The network can be visualized using a wide variety of applications, including **a** CerebralWeb, **b** tabular format, **c** Cytoscape, **d** the Cerebral Java Webstart, **e** Biolayout, **f** CyOog or **g** NetworkAnalyst

The network generated using InnateDB can be interactively visualized using a variety of different visualization tools (Fig. 2). The InnateDB network analysis result page provides an embedded visualization of the network using the CerebralWeb application [31], which lays out the nodes in layers based on their subcellular localization. Below this, a tabular text-based description of each interaction in the network is presented, which contains further links to more detailed information for each interaction. In addition, interaction networks may be visualized and analyzed in Cerebral [32], a Java webstart plugin for the Cytoscape network visualization software [33]. Networks can also be investigated via other third-party software, including the CyOog plugin [34], which uses Power Graph analysis to reduce network complexity, and BioLayout Express 3D 2.2 [35], which is designed to visualize large networks in 2D and 3D space. All three applications are available via the “*Visualization*” menu, which is located beneath the main embedded network visualization. From here, one can also transfer the uploaded gene list to NetworkAnalyst [36] for further network analysis (see below). All networks that are constructed using InnateDB can be downloaded in several standard formats, including text-based (.tab, .csv, .xls), the *simple interaction format* (.sif), Cytoscape’s XGMML format, and both the PSI-MI XML 2.5 and MITAB exchange formats [37]. We recommend downloading the XGMML format (http://wiki.cytoscape.org/XGMML), which not only contains information on the nodes and edges in the network, but also their associated attributes and information on how to graphically represent and lay out the network. This format can be readily imported into Cytoscape for further analysis and to harness the diverse range of third party Cytoscape Apps that are available.

## Inferring biologically important properties/features from networks

Constructing a network, while important, is only the first step of any network analysis. Without further investigation of network features (e.g. node degree or network modularity) and how these features potentially deviate from statistical expectation, building a network does little more than generate a pretty (or sometimes ugly) picture. Fortunately, numerous mathematical and computational approaches have been developed to analyze large networks to identify features of interest.

### Network hubs

**Fig. 3:**
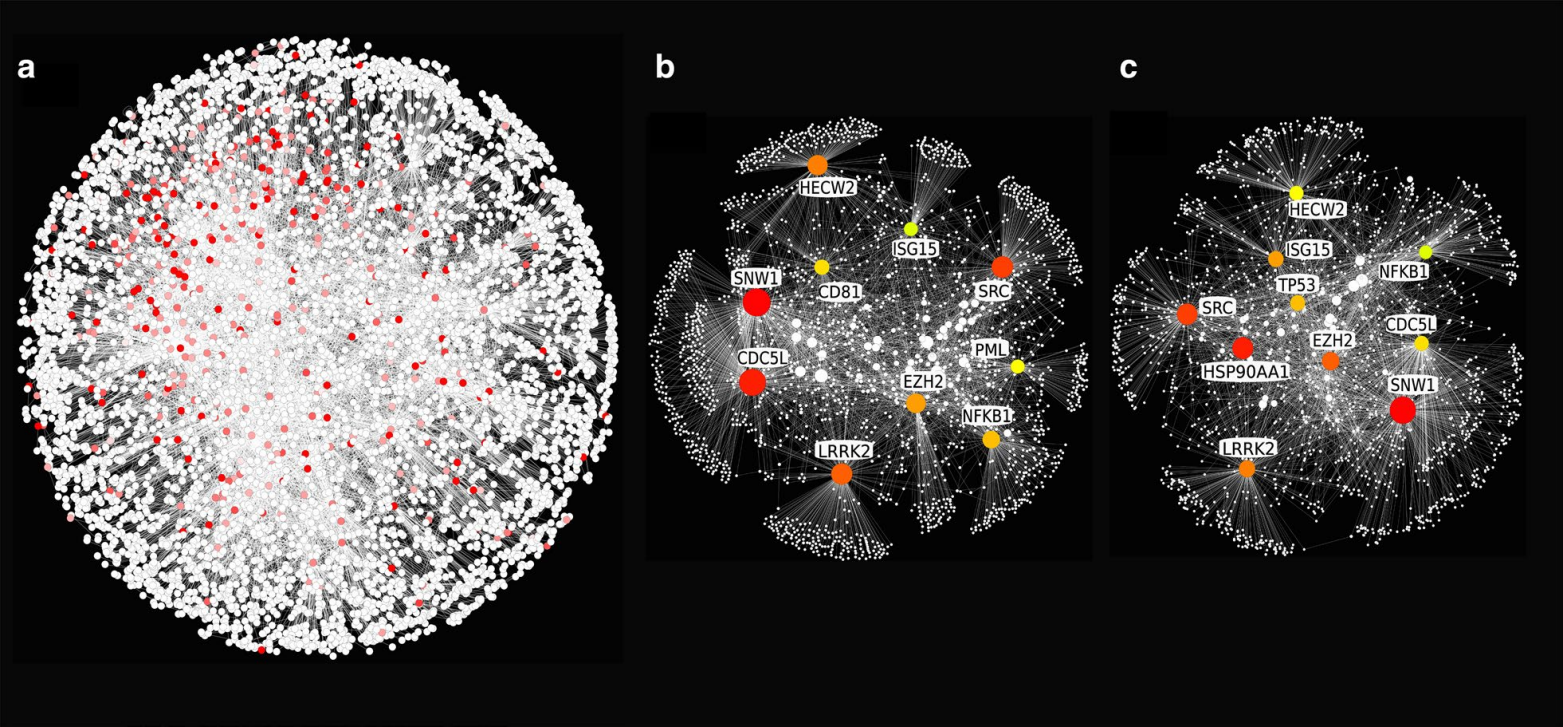
Network hubs and bottlenecks. **a** InnateDB was used to construct a network of genes that were significantly up-regulated in the Lawless et al. [30] dataset. Interactions between the genes in the uploaded list, as well as all their interacting partners, were included in the network. The network consisted of 6259 nodes and 15,137 edges (self-loops; duplicated edges; and edges involving UBC were removed). **b** Hub nodes were identified using the CytoHubba plugin in Cytoscape 2.8.2. Cytoscape was used to extract and visualize the top 10 hub nodes and their interactors. Hub nodes are shown in *colour* with gene names. Node size is proportional to degree. **c** CytoHubba was also used to identify bottleneck nodes in the network. The top 10 bottleneck nodes are shown in *colour* with gene names. There is considerable overlap between the top 10 hub nodes and the top 10 bottleneck nodes

One feature that is often informative in network analysis is node *degree* (i.e. number of interactions/edges/connections that a node has). Molecular interaction networks generally exhibit a scale-free topology, where the degree distribution approximates a power law and in which most nodes have few edges and a small number of nodes have a very high degree [38]. These high degree nodes are termed *hubs* (Fig. 3). Hub nodes are topologically important to the network structure and are often functionally important. The deletion of a hub gene, for example, is more likely to be lethal than the deletion of a non-hub gene (*Centrality*-*lethality rule*) [39], although the exact reasons for this correlation are still being debated [40–42]. Scale-free networks are also more robust to random failures than more uniform degree distributions but are more susceptible to targeted attacks [43]. Indeed, hub proteins have repeatedly been found to be preferentially targeted by pathogens [44–46]. It has also been suggested that genes encoding hub proteins are enriched for disease genes. Several studies have shown that cancer-related genes tend to be more highly connected than expected [47, 48], although more careful consideration of the biases in PPI networks suggests that this may only be the case for particular types of cancers [49]. Finally, because hubs participate in many interactions, they are more likely to be master regulators of signalling and transcription. For example, the hub proteins uncovered in the transcriptional response networks in bovine macrophages differ between infections with virulent versus avirulent *Mycobacterium bovis*, the causative agent of bovine tuberculosis [50].

### Network bottlenecks

The distance between two nodes in a network can be measured by determining the minimum number of steps between them [51]. Bottleneck nodes are defined as nodes with a high *betweenness centrality* (i.e. network nodes that have many “shortest paths” going through them) [52]. Bottleneck nodes play key roles in mediating communication within a given network because they facilitate information flow between modules (relatively densely connected sub-networks, see next section). Such nodes are therefore like chokepoints in the network and have been described as being analogous to major bridges and tunnels on a highway map [52]. Disruption of a bottleneck can lead to network “traffic” chaos, since there are few or no alternative routes around the bottleneck. Bottleneck nodes have been found to be more highly correlated with essentiality than hub nodes [53] and are also preferentially targeted by pathogens [44, 45]. It should be noted that the top hub and bottleneck nodes often tend to be very similar (Fig. 3). Lawless et al. [30], for example, constructed a network of genes that were differentially expressed in monocytes isolated from milk at 36 h post-infection with *S. uberis* and showed that 85 % of the top 20 hub proteins in the network were also bottleneck nodes. Thus, it can often be difficult to assess whether a node is important because it is highly connected or because it is a bottleneck.

### Network modules

**Fig. 4:**
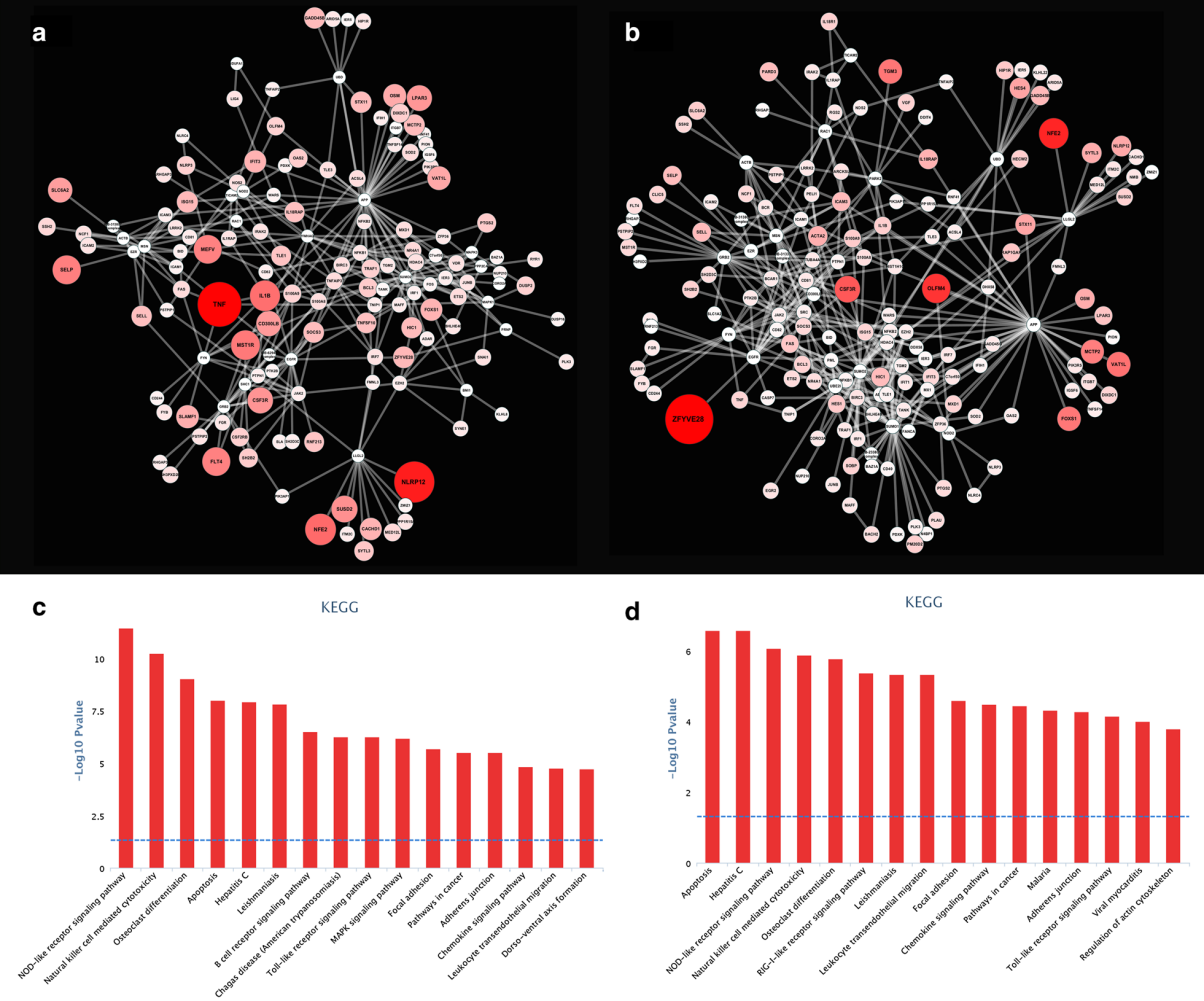
Identifying modules in a network. The *jActiveModules* plugin in Cytoscape 3.1.0 was also used to identify high-scoring differentially expressed (DE) sub-networks in the network of Fig. 3a (using parameter values: overlap threshold = 0.3; search depth = 2). The highest-scoring module identified in the network using **a** the gene expression data at 36 h post-infection (hpi) and **b** at 48 hpi, are shown. The InnateDB pathway analysis tool was used to identify over-represented pathways for **c** the 36 hpi module and **d** the 48 hpi module. Note that for this dataset, the top five pathways for the 36 and 48 hpi modules are very similar, which reflects a similar pattern of gene expression at these time-points in this dataset

Another important feature of many molecular networks is that they are modular in nature and have a high *community* structure [54]. Genes or proteins that occur in particular modules tend to be enriched for common biological functions [55]. Thus, identifying modules in networks can identify coordinated biological functions or processes that are not well captured in established canonical pathway annotations. Proteins that are involved in the same disease or in diseases with similar phenotypes have also been shown to preferentially interact with each other in “disease modules” [15]. One can therefore identify network modules that are enriched in genes/proteins known to be associated with a disease of interest (Fig. 4). Other proteins in these modules, which are not currently known to be associated with the disease, are promising disease-associated candidate genes. This network module approach has now been widely implemented to identify disease-associated modules for a range of human diseases. For example, 39 % of *de novo* severe or disruptive mutations that were associated with autism through exome sequencing, were found to map to a highly interconnected β-catenin/chromatin remodelling module [56]. Similarly, risk factors for congenital heart disease have been shown to functionally converge in network modules that regulate heart development [57]. These approaches are also gaining popularity in animal genomics studies, particularly in the identification of functionally relevant sub-networks from gene co-expression networks [58–60].

### Bioinformatics apps to identify hubs, bottlenecks and modules

A wide variety of bioinformatics tools to quickly identify network hubs and bottlenecks are available. Some examples include the aforementioned *NetworkAnalyst*, a tool to support network-based gene expression meta-analyses [36]. *NetworkAnalyst* imports a list of user-defined genes and associated interactions from InnateDB to calculate degree, betweenness centralities and functional modules in the network (see below for further discussion of network modules). The Cytoscape platform also provides an ecosystem of mainly third party *Apps* that can be used to undertake these and more advanced network analyses [33]. One such App is *cytoHubba,* which can be used to identify hubs and bottlenecks in networks imported into Cytoscape [61]. This can be used in conjunction with networks that are generated by using InnateDB, which can be downloaded in XGMML format and then imported into Cytoscape.

A variety of computational tools have also been developed to identify modules in networks. For a comprehensive review, we refer the reader to [62]. Here we introduce some useful tools that represent a good starting point for a researcher who is new to this topic. *NetworkAnalyst* also contains more advanced network analysis features that can be used to identify potentially functionally relevant network modules. *NetworkAnalyst* uses a random walk algorithm to identify modules of frequently visited nodes. It can also generate an edge weighted network, in which weights are derived from quantitative node information, such as gene expression attributes [63]. Cytoscape also provides a number of user-friendly applications for module detection, including *jActiveModules* [64], which identifies connected regions of a network that also show significant changes in gene expression.

However, if the aim is to find disease-associated modules, other algorithms may perform better, since it was recently reported that disease-associated proteins do not reside in particularly dense local communities and that disease-related nodes may be better predicted using *connectivitysignificance* (i.e. whether the number of connections from a candidate protein to other known disease “seed” proteins is greater than statistically expected by chance) [65]. The *Disease Module Detection algorithm* (DIAMOnD) is a new method to detect disease modules based on connectivity significance.

Apart from the choice of a network analysis tool, researchers need to be aware that the incompleteness of the interactome limits which disease modules can be detected, and that there is a minimum threshold for the number of known disease-associated proteins to be able to detect modules associated with a disease of interest [66]. Finally, it should be noted that the detection of hubs, bottlenecks and modules is only the tip of the iceberg when it comes to network analyses and further analyses should be driven by the research questions that are specific to each study.

## Conclusions and further discussion

In this review, we introduce network analysis and show that it is a powerful tool to assist researchers in the interpretation, visualisation, and analysis of genome-wide ‘omics’ data. However, significant challenges remain to be addressed. Unlike mapping the genome of a species (although the genome can also vary considerably between individuals), mapping the protein interactome of a species is something of a fallacy. The interactome is a highly dynamic entity that depends on the temporal, spatial, cellular and environmental contexts. Fortunately, with advances in technology, we are now moving towards an era of dynamic interactome studies [67]. Recently, for example, researchers have mapped the Hippo signalling pathway protein–protein interaction network in the presence and absence of inhibition by serine and threonine phosphatases, and revealed how changes in phosphorylation result in a significant re-wiring of the protein interactions between members of this pathway [68]. Similarly, Jäger et al. [69] have systematically determined the physical interactions of all 18 HIV-1 proteins and polyproteins with host proteins in two human cell lines (HEK293 and Jurkat) and showed that only about 40 % of interactions occurred in both cell types, which provides insight into just how different the interactome is likely to be in two different cell types. PPI networks are also likely to be substantially re-wired in diseases, with the effect of any given mutation rippling through the network and causing a re-wiring of proteins that otherwise carry no defects [15]. Indeed, a recent study has shown that perturbation of protein–protein interactions is widespread in human genetic disorders [70]. Investigating thousands of missense mutations, Shani et al. [70], showed that two-thirds of disease-associated alleles perturb protein–protein interactions.

Network re-wiring in different contexts will also change which topological and functional network features are important. Network re-wiring will likely have an impact on the top hub and bottleneck proteins, e.g. a hub node in a normal network may be less central in a disease-associated network and vice versa. Such re-wiring may also have an impact on the set of network modules that are identified in a disease network or in another phenotype of interest. Thus, an important focus for network biology will be to experimentally reconstruct and compare networks in normal and disease conditions to determine network features or components that are specifically associated with disease [71]. An interesting future direction here is the question of how to target disease-associated networks for destruction while preserving normal network function [72].

Similarly, it will also be of significant interest to computationally model how network re-wiring may have an impact on how signals flow through the network and alter network outputs, such as the activation of differing transcriptional responses. Several approaches have been proposed to investigate how signals flow through large biological networks, in particular protein–protein interaction (PPI) networks, for which substantial amounts of data are publicly available [73, 74]. One promising approach is *information flow analysis*, a computational biology method that uses random walk algorithms to model how signals flow through large networks. One example of software that performs this type of analysis is *ITM Probe* [75], which is also available as a Cytoscape App [76]. In ITM Probe, the user can define source nodes (nodes that *emit* information, e.g., receptors) and sink nodes (target nodes that *absorb* information, e.g., transcription factors). The algorithm then models information flow in a protein interaction network through discrete time random walks, where the walker has a certain probability to dissipate (i.e. to leave the network) at each step. Edge weight and interaction direction information can also be used to assign higher probabilities to certain paths through the network than others. The more times random walkers pass through a node, the higher the information flow score for that node will be. By altering the network between the source and sink nodes, one can computationally infer the impact of network re-wiring on information flow in the network.

While experimentally reconstructing networks under different conditions is an important goal, this will remain costly and technically challenging for most research groups well into the future. Fortunately, by overlaying dynamic data that is more readily generated (e.g. gene expression data) onto experimentally-validated networks (e.g. PPI), one can already gain insight into which network features might be preferentially associated with disease or another phenotype of interest. For example, a static map of the interactome can show some hub nodes with large numbers of connections. However, proteins have a limited number of structural interfaces with which to engage in direct protein–protein interactions and cannot interact directly with so many partners at the same time [77]. This has led to the classification of hubs as either “*party*” hubs, which interact with most of their partners simultaneously, or “*date*” hubs, which bind their different partners at different times or locations [78], although this classification remains hotly debated [79, 80]. Regardless of whether this is a useful classification or not, it is clear that if one takes multiple random lists of genes and builds a network, one will find that some nodes are always or frequently identified as hubs because they are highly connected in the database and not necessarily because they are relevant to the condition of interest. Therefore, it is important for the researcher to calculate statistical significance based on this background expectation (e.g. using a hypergeometric distribution test), in a manner similar to that described previously for functional enrichment analysis.

In conclusion, networks provide a powerful conceptual approach to integrate and find patterns in genome-wide genomics data but researchers adopting these approaches need to be conscious of their limitations and caveats. In this review, we have mainly focused on PPI networks but a wide variety of other types of networks are becoming ever more prevalent in the scientific literature, including gene co-expression networks, transcriptional regulation networks, and metabolic networks [71, 81]. The great challenge will be to integrate these various types of networks into a universal network model of the cellular interactome.

## Additional file

10.1186/s12711-016-0205-1 Case study gene expression data. Description: 514 genes from Lawless et al. [30] that were found to be significantly up-regulated more than threefold in monocytes isolated from milk at either 36 or 48 h post-infection (hpi) with the pathogen *Streptococcus uberis* that causes mastitis in cattle.
